# Factors involved in patients’ perceptions of self-improvement after chronic pain treatment

**DOI:** 10.1080/24740527.2018.1476821

**Published:** 2018-06-18

**Authors:** Majidullah Shaikh, Eleni G. Hapidou

**Affiliations:** a Department of Psychology, Neuroscience and Behaviour (PNB), McMaster University, Hamilton, ON, Canada; b Michael G. DeGroote Pain Clinic, McMaster University Medical Centre, Hamilton, ON, Canada; c Michael G. DeGroote Institute for Pain Research and Care, McMaster University, Hamilton, ON, Canada; d Department of Psychiatry and Behavioural Neurosciences, McMaster University, Hamilton, ON, Canada; e Bachelor of Health Sciences (Honour’s) Program, McMaster University, Hamilton, ON, Canada

**Keywords:** chronic pain, self-improvement, pain management, self-evaluation

## Abstract

**Background:**

Patients’ self-reported levels of improvement after having attended a chronic pain management program can provide a subjective rating of how successful they perceive they were at accomplishing their goals in the program. Past studies have demonstrated that successful patients differ from less successful ones on several cognitive–behavioral factors such as coping strategies over physical characteristics such as pain intensity.

**Aims:**

This study explored factors that determine patients’ perceptions of self-improvement after undergoing chronic pain treatment in a pain management program.

**Methods:**

Participants (*n* = 174) underwent a 4-week, interdisciplinary, multimodal, chronic pain management program at a hospital located in southern Ontario. Questionnaire packages that evaluate pain intensity, pain-related disability, emotional distress (e.g., depression, anxiety, catastrophizing), acceptance of pain (activity engagement and pain willingness), readiness to change, and use of adaptive or maladaptive coping strategies were completed by patients at admission and discharge. Participants were grouped into one of three categories depending on their rating of self-improvement on the Self-Evaluation Scale (SES). The groups were compared on the magnitude of change they reported on the variables mentioned above.

**Results:**

Changes in emotional distress, general health, readiness to change, activity engagement, and adaptive coping strategies (e.g., task persistence, pacing, and seeking social support) were significantly associated with differences in ratings of self-improvement.

**Conclusions:**

This study provided insight into what patients value most when rating their self-improvement, which can then be used to facilitate increased patient success and satisfaction with treatment.

## RÉSUMÉ

**Contexte** : Le niveau d’amélioration autodéclaré par les patients après avoir participé à un programme de gestion de la douleur chronique peut fournir une évaluation subjective du succès qu’ils perçoivent avoir obtenu pour atteindre leurs objectifs dans le cadre de ce programme. Des études effectuées par le passé ont démontré que les patients qui ont eu du succès différent de ceux qui ont eu moins de succès en ce qui concerne plusieurs facteurs cognitivo-comportementaux comme les stratégies d’adaptation, plutôt que des caractéristiques physiques comme l’intensité de la douleur.

**But** : Cette étude portait sur les facteurs qui déterminent les perceptions des patients en ce qui concerne l’amélioration de leur état après avoir été soumis à un traitement pour la douleur chronique dans le cadre d’un programme de gestion de la douleur.

**Méthodes** : Les participants (n = 174) ont participé à un programme de gestion de la douleur chronique interdisciplinaire et multimodal d’une durée de quatre semaines dans un hôpital situé dans le Sud de l’Ontario. Au moment de leur admission au programme et au terme de celui-ci, les patients ont répondu à des questionnaires évaluant l’intensité de la douleur, l’incapacité liée à la douleur, la détresse émotionnelle (ex.: dépression, anxiété, dramatisation), l’acceptation de la douleur (engagement dans des activités et volonté de ressentir la douleur), la disposition au changement et l’utilisation de stratégies pour s’adapter ou ne pas s’adapter. Les participants ont été répartis en trois catégories selon l’évaluation qu’ils faisaient de l’amélioration de leur état à l’aide d’une échelle d’autoévaluation. L’ampleur du changement déclaré par chacun des groupes a été comparé pour chacune des variables susmentionnées.

**Résultats** : Les changements dans la détresse émotionnelle, la santé générale, la disposition au changement, l’engagement dans des activités et les stratégies d’adaptation (ex. : la persistance dans les tâches, le rythme et le recours à du soutien social) ont été associés de manière significative à des différences dans l’évaluation de l’amélioration personnelle.

**Conclusions** : Cette étude a permis de mieux comprendre ce que les patients valorisent le plus lorsqu’ils évaluent l’amélioration de leur état, ce qui peut par la suite être utilisé pour accroître le succès du patient et sa satisfaction à l’égard du traitement.

**Abbreviations** PSOCQ: Pain Stages of Change Questionnaire; CPAQ: Chronic Pain Acceptance Questionnaire; PIS: Pain Intensity Scale; PDI: Pain Disability Index; PCS: Pain Catastrophizing Scale; PRIME-MD PQ: Patient Questionnaire of the Primary Care Evaluation of Mental Disorders; CAS: Clinical Anxiety Scale; CES-D: Center for Epidemiological Studies–Depressed Mood Scale; CPCI: Chronic Pain Coping Inventory; SES: Self-Evaluation Scale

Chronic pain is any persistent pain that lasts longer than 3–6 months or longer than the duration of time it would normally take for the body to heal.^1^ People who have been living with pain for a long time often report feelings of helplessness, loneliness, anxiety, depression, low self-efficacy, and low self-esteem.^2,3^ Chronic pain management programs help patients adopt a self-management approach to handling their chronic pain. This approach could then help to improve functioning and adjustment for patients; reduce negatively associated symptoms, beliefs, and behaviors; and help patients to resume their typical daily activities. To help design and facilitate these programs, it is important to gain an understanding of what factors patients find most important to attribute their treatment as a success.

Traditionally, chronic pain management programs combine cognitive–behavioral therapy (CBT), which addresses the dysfunctional emotions and maladaptive behaviors associated with chronic pain, with physical therapy, which addresses the physical factors of pain such as restoring mobility, enhancing physical fitness, and remediation of movement. CBT techniques combined with physical therapy tend to show more benefit than physical therapy alone.^4–6^

Previous literature has evaluated the success of chronic pain management as an effective intervention for improving quality of life and reducing negative pain-related outcomes. In a meta-analysis by Morley et al.,^7^ patients who underwent CBT improved more than waitlist controls on factors of pain experience, mood and affective state, use of positive coping strategies, avoidance of negative coping strategies, avoidance of negative pain-related behaviors, and social role functioning. In another meta-analysis of chronic pain management programs conducted by Morley et al.,^8^ significant reductions were observed in pain interference, pain intensity, pain distress, psychological distress (depression and anxiety), and catastrophizing, whereas higher levels of walking and self-efficacy were observed. Finally, in multiple studies conducted by Hapidou and colleagues,^9–12^ patients who attended a 4-week program reported higher levels of acceptance of pain (in terms of both activity engagement and pain willingness), self-management approaches to pain that involve taking action and maintenance of pain management, and adaptive coping strategies such as exercise/stretch, relaxation, pacing, seeking social support, and coping self-statements after treatment. In addition, patients reported lower levels of pain intensity, pain-related disability, emotional distress (e.g., depression, catastrophizing, anxiety), health problems, and maladaptive coping strategies such as guarding, resting, and asking for assistance. However, it is important to note that recent reviews of chronic pain management programs involving CBT in the treatment of chronic pain only yielded small to moderate effects sizes (Cohen’s *d* ranging from 0.2 to 0.5).^13^ Therefore, there is room to improve such programs in order to increase their positive effects on patient outcomes.

In traditional treatment models, the criteria for patient success are determined by health care providers; however, outcomes that may be important to providers may not match the outcomes that patients find important.^14^ A recent shift toward patient-centered treatment has grown in the last decade, which involves more collaboration between health care providers and patients, with providers gaining an understanding of patients’ needs and taking a more individualized approach to help these patients reach their goals. A patient-centered model of treatment has been associated with increases in patient satisfaction with health care, adherence to treatment, and stronger and more sustained patient–provider relationships, in comparison to the traditional medical model of treatment.^15,16^ However, more research is needed to understand the factors that patients find important in their journey to improvement, in order to inform patient-centered treatment.

To help gain an understanding of patients’ needs, it is important to capture what constitutes self-improvement (defined as positive self-perceptions of success, performance, or goal achievement) for patients. For instance, measures that evaluate a patient’s global impression of changes have been found to be useful in identifying the extent to which pain-related variables (e.g., pain intensity) needed to be reduced/improved in order to identify a clinically important outcome after chronic pain trials.^17,18^ Patients’ ratings of success and improvement have been identified as core outcome domains that should be considered by providers in chronic pain management programming, based on the IMMPACT recommendations.^19^

Within chronic pain management contexts that involve psychosocial therapies, previous literature has explored factors that are valued by patients in their judgments of overall self-improvement. O’Brien et al.^16^ found that for patients to rate their treatment as successful, they required at least 54%–58% reduction in pain intensity, 60%–67% reduction in distress (e.g., depression, anxiety), and 63%–68% reduction in pain interference (e.g., pain-related disability). Fisher^20^ found that higher ratings on the Goal Attainment Scale (GAS), a 5-point scale measuring patient-generated perceptions of their goal achievement, were associated with higher levels of improvement in pain intensity, pain-related disability, general health, and exercise. Furthermore, Kerns and Rosenberg^21^ found that patients’ positive ratings of goal achievement were associated with improvements in readiness to change; in particular, this involved decreases in precontemplation (i.e., beliefs that pain relief is primarily the responsibility of physicians and medical treatment) and increases in action (i.e., the acceptance of a self-management approach and engagement in such treatment) and maintenance (i.e., intention to continue a self-management approach). These studies provide initial insights on factors that may be involved in patients’ perceptions of their success.

Though the preceding paragraph outlined initial evidence on what patients value in their chronic pain treatment, given the interdisciplinary and multimodal nature of chronic pain management programs, more research is necessary to examine the association of various additional psychosocial factors on judgments of self-improvements. For instance, little research has explored how improvements in use of approaches such as pain acceptance, adaptive coping strategies, and reduction in maladaptive coping strategies may be associated with patients’ ratings of global self-improvement. This warrants further study because these factors have all been associated with positive functioning in patients.^22^ Additionally, little research has explored the nuance between what makes patients who rate their improvement as successful different from those who rate their improvement as poor. Insight into this issue can help providers understand the unmet needs for patients who differ in their ratings of self-improvement.

## Research questions

The purpose of this study was to understand the psychosocial factors associated with patients’ perceptions of self-improvement. Improvement in this study was understood as the extent to which a chronic pain management program helped patients accomplish their goals, which included improving fitness, reducing medications, improving general health and nutrition, increase participation in family and social life, and improve functioning at work. The psychosocial factors examined were those recognized from previous literature as important to patients’ ratings of self-improvement as well as those that are yet to be explored. As such, it was predicted that patients who report higher levels of global self-improvement will also report significantly greater improvements on psychosocial factors of pain acceptance (e.g., activity engagement, pain willingness), stages of change (e.g., precontemplation, action, and maintenance), and adaptive coping strategies (e.g., exercise/stretch, relaxation, pacing, seeking social support, and coping self-statements) while reporting significantly greater reductions in pain intensity, emotional distress (e.g., depression, anxiety, and catastrophizing), health problems, pain-related disability, and maladaptive coping strategies (e.g., guarding, resting, and asking for assistance) after treatment, relative to those patients who reported poorer levels of self-improvement.

## Method

### Procedure

Ethics approval for this study was obtained from an institutional ethics review board. The data analyzed in this study were collected from patients admitted to a southern Ontario hospital between January 2010 and December 2012. Patients had experienced chronic pain following a work-related injury or motor vehicle accident and were referred to the hospital by the Workers Safety and Insurance Board, their insurance company, or their lawyer. After consent was obtained, each patient completed a package of self-report measures consisting of a demographic form as well as 9 initial questionnaires: the Pain Intensity Scale (PIS),^23^ Center for Epidemiological Studies–Depressed Mood Scale (CES-D),^24^ Pain Catastrophizing Scale (PCS),^25^ Clinical Anxiety Scale (CAS),^26^ Patient Questionnaire of the Primary Care Evaluation of Mental Disorders (Prime-MD PQ),^27^ Pain Disability Index (PDI),^28^ Chronic Pain Acceptance Questionnaire (CPAQ),^29^ Pain Stages of Change Questionnaire (PSOCQ),^30^ and Chronic Pain Coping Inventory (CPCI).^31^ These patients then attended a 4-week, multimodal, interdisciplinary chronic pain management program. At discharge, each patient completed a package that included the same measures mentioned above, along with an additional measure, the Self-Evaluation Scale (SES).^9^

### Participants

Participants consisted of 174 patients who completed the 4-week program and had complete program evaluation data at both admission and discharge. Of this sample, 83 were males and 87 were females (data on sex were not available from two people), between the ages of 25 and 64 years (M = 45.85, SD = 9.09). The majority of the sample were born in Canada (72.4%), were married (53.4%), were unemployed (63.8%), and had sustained injury through a motor vehicle accident (35.1%) or work-related accident (28.7%). On average, participants had been off work for 35.87 months (SD = 47.28), had 13.36 years of education (SD = 3.73), had pain for 54.72 months (SD = 48.85), and had sustained at least two injuries. Participants were taking medications such as opioids, anti-depressants, anti-inflammatory drugs, and sleep medications.

### Measures

#### Demographics

Each participant was asked to provide information about his or her age, marital status, education level, employment status, and the number of years he or she had resided in Canada. Each participant also included specific details about the type of injury, including the source, pain duration(s), and number of injuries that he or she had sustained.

#### Pain Intensity Scale

The participant reports his or her “usual” and “least” subjective levels of pain experienced in the past 2 weeks.^23^ This 11-point scale ranged from 0 (*no pain*) to 10 (*unbearable pain*). The PIS has been shown to be a valid measure of pain intensity through its strong association with the PDI and other pain measures.^10,32–34^ The PIS has demonstrated high reliability in comparison to visual and verbal measures and high responsiveness to change.^35,36^

#### Centre for Epidemiological Studies–Depressed Mood Scale

The CES-D was used to assess symptoms of depressive state experienced by the participant in the past week.^24^ This measure consists of 20 questions on a 4-point scale ranging from 0 (*rarely or none of the time; less than 1 day*) to 3 (*most or all the time; 5–7 days*). The CES-D’s criterion validity has been shown, because its scores were positively correlated with other self-report scales that measure symptoms of depression (*r* = 0.55–0.74) convergent validity; and negatively correlated with scales measuring variables different from depression (*r* = −0.55) discriminant validity. Evaluated test–retest reliability of the CES-D has found moderate correlations (*r* = 0.45–0.7) between initial and follow-up scores 3 to 12 months after the initial questionnaire was given.^24^

#### Pain Catastrophizing Scale

The PCS was used to assess the participant’s level of catastrophizing; the participant rates how frequently he or she perceives feelings related to rumination, magnification, and helplessness as a result of his or her pain experience.^25^ There are 13 items on a 5-point scale, which ranged from 0 (*not at all*) to 4 (*all the time*). The PCS has demonstrated convergent validity, as evidenced by the moderate correlation of total PCS scores with scores on negative affectivity (*r* = 0.75, *P *< 0.001) and self-reported anxiety measures (*r* = 0.32, *P *< 0.001). The PCS has demonstrated acceptable and satisfactory internal consistency for total score (α = 0.97) and its three subscales (i.e., Rumination) (α = 0.87), Magnification (α = 0.60), and Helplessness (α = 0.79), and strong test–retest reliability has been established for 6 weeks (*r* = 0.75) and 10 weeks (*r* = 0.70) in a sample population.^25^

#### Clinical Anxiety Scale

This CAS was used to assess the participant’s level of state anxiety.^37^ The measure consists of 25 items on a 5-point scale, which range from 1 (*rarely or none of the time*) to 5 (*most or all of the time*). The CAS has demonstrated good discriminant validity (*r* = 0.77), effectively distinguishing between low-anxiety and clinical anxiety groups. The CAS has been shown to be a very reliable measure indicated by a high internal consistency (α = 0.94) and low standard error of measurement (4.2).^35^

#### Patient Questionnaire of the Primary Care Evaluation of Mental Disorders

The PRIME-MD PQ was used to assess the participant’s physical and emotional symptoms experienced in the past month.^27^ The scale contains 25 true or false questions, followed by a 5-point rating of the patient’s self-perceived health, as either “excellent,” “very good,” “good,” “fair,” or “poor”. The scale has demonstrated excellent overall accuracy (88%) and good agreement (κ = 0.71). In addition, the PRIME-MD PQ has been shown to be a useful tool in screening mental disorders, demonstrating good to excellent sensitivity across all diagnoses, including mood (69%), anxiety (94%), alcohol (81%), and eating (86%) disorders.^27^

#### Pain Disability Index

The PDI was used to assess the extent of disability to which the participant attributes his or her chronic pain condition.^28^ The measure consists of seven questions on an 11-point scale, with a range from 0 (*no disability*) to 10 (*total disability*), to rate the participant's experience in each of the seven categories of life activities: family/home responsibilities, recreation, social activity, occupation, sexual behavior, self-care, and life support activity. The construct validity of the PDI has been established, because patients with higher PDI scores had significantly more pain characteristics, including restriction of activities and psychological distress (all *P *< 0.001), than patients with low PDI scores. The PDI has been shown to be a reliable measure, demonstrating high internal consistency (Cronbach’s α = 0.86) and high test–retest reliability (intraclass correlation coefficient = 0.91) in patients who repeated the questionnaire 1 week after its initial completion.^32,38^

#### Chronic Pain Acceptance Questionnaire

The 20-item CPAQ was used to assess the degree of acceptance that a participant had for his or her chronic pain condition.^39^ The CPAQ has two subscales: Activity Engagement and Pain Willingness. The participant rates each statement on a 7-point scale, which ranges from 0 (*never true*) to 6 (*always true*). The CPAQ has demonstrated adequate predictive validity, because outcomes like depression, pain-related anxiety, and psychosocial disability could be significantly predicted by both Pain Willingness (all *P *< 0.05) and Activity Engagement (all *P *< 0.05) subscales. The CPAQ has demonstrated good internal consistency for Activity Engagement (α = 0.82) and Pain Willingness (α = 0.78) subscales.^29,39^

#### Pain Stages of Change Questionnaire

The PSOCQ was used to assess the participant’s readiness to change and adopt coping strategies taught by the clinicians for his or her condition.^30^ The participant rated how strongly he or she agreed or disagreed with statements using a scale from 1 (*strongly disagree*) to 5 (*strongly agree*).^30^ This questionnaire includes four subscales measuring the four stages of change: Precontemplation, Contemplation, Action, and Maintenance^30^ The PSOCQ has demonstrated criterion-related validity, because measures of control, accommodation, and active coping were positively related to maintenance (*r* = 0.61, *r* = 0.52, *r* = 0.49, respectively) and negatively related to precontemplation (*r* = −0.55, *r* = −0.37, *r* = −0.35, respectively).^30^ Excellent reliability has been established in each subscale (i.e., Precontemplation) (α = 0.77), Contemplation (α = 0.82), Action (α = 0.86), and Maintenance (α = 0.86), and excellent test–retest reliability has been shown (α = 0.74–0.88 over a 1- to 2-week period).^30^

#### Chronic Pain Coping Inventory

The CPCI was used to assess the participant’s use of coping strategies, with statements that evaluate how many days in the past week he or she performed certain actions to deal with pain.^31^ The measure consists of nine coping strategies presented on nine subscales, six of which were adaptive strategies and were encouraged, whereas the other three were maladaptive and discouraged.^31^ The adaptive strategies are exercise/stretch, relaxation, task persistence, coping self-statements, pacing, and seeking social support, and the maladaptive strategies are guarding, resting, and asking for assistance.^31^ The validity of this measure has also been established and can significantly predict pain adjustment (e.g., pain severity, interference, negative emotion, self-control, and social support).^40^

#### Self-Evaluation Scale

The SES was used to determine the participant’s self-perceived performance in the program. The SES^9^ asks, “To what extent do you think you have accomplished your goals in the past 4 weeks?” It was used to determine the patient’s perceived goal accomplishment at the end of the 4-week interdisciplinary chronic pain management program. It was scored using a 5-point scale: 1 (*poorly*), 2 (*fairly*), 3 (*well*), 4 (*very well*), and 5 (*excellent*). This was followed by an open-ended section titled “Comments” where the patient could elaborate on his or her perceived goal accomplishment in various areas of functioning. The SES was found to be reliable and valid in assessing goal accomplishment^9,12^ in a multidisciplinary chronic pain management program. It has also been previously used in a study on kinesiophobia^41^ as a reference standard to dichotomize participants into two groups: those who scored 3, 4, or 5 on the SES at discharge were categorized as *having an important reduction* in their fear of movement/reinjury, and those who scored 1 or 2 were categorized as *not having an important reduction* in their fear of movement/reinjury.

### Data analysis

The quantitative data were analyzed using SPSS (IBM SPSS Statistics for Windows, Version 23.0, Armonk, NY). Participants were divided into three groups based on their ratings on the SES. Those who rated themselves 1 or 2 were placed in the “poorly–fairly” group, those who rated themselves 3 were placed in the “well” group, and those who rated themselves 4 or 5 were placed in the “very well–excellent” group. The poorly–fairly group represented perceptions of improvement that were unfavorable in terms of program outcomes, the well group represented perceptions that were moderate, and the very well–excellent group represented those perceptions that were most favorable. To evaluate whether there was an association between psychosocial factors and self-reported improvement between rating groups, a mixed analysis of variance (ANOVA) was conducted using SPSS. This compared the mean scores of multiple dependent variables (e.g., the psychosocial factors) with a within-subjects factor (time) and a between-subjects factor (rating groups). A conservative alpha level of 0.002 (0.05/21) was employed to address the use of multiple tests. Post hoc tests with a Bonferroni correction were performed on relationships that demonstrated significant differences.

## Results

Demographic frequencies and descriptive statistics were gathered for each of the three rating groups and are displayed in Table 1. At baseline, age was the only demographic factor in which a difference between rating groups was found; the very well–excellent was significantly older than the well group (M_diff_ = 4.41, *P* = 0.007).

**Table 1.** Demographic statistics of participants.

	Poorly–fairly *n* (%)		Well *n* (%)		Very well–excellent *n* (%)		Total *n* (%)	
Number of participants	34 (19.5)		60 (34.5)		80 (46.0)		174 (100)	
Gender								
Male	15 (18.1)		27 (32.5)		41 (49.4)		83 (47.7)	
Female	18 (20.7)		32 (36.8)		37 (42.5)		87 (50.0)	
Unlisted	1 (25.0)		1 (25.0)		2 (50.0)		4 (2.3)	
Born in Canada								
Yes	24 (19.0)		43 (34.1)		59 (46.8)		126 (72.4)	
No	7 (29.2)		8 (33.3)		9 (37.5)		24 (13.8)	
Unlisted	3 (12.5)		9 (37.5)		12 (50.0)		24 (13.8)	
Marital status								
Married/common-law	20 (21.5)		30 (32.3)		43 (46.2)		93 (53.4)	
Single	5 (16.1)		14 (45.2)		12 (38.7)		31 (17.8)	
Separated/divorced/widowed	6 (21.4)		7 (25.0)		15 (53.6)		28 (16.1)	
Unlisted	3 (11.5)		9 (34.6)		14 (53.8)		26 (14.9)	
Employment Status								
Unemployed	26 (23.4)		39 (35.1)		46 (41.4)		111 (63.8)	
Employed	5 (12.8)		11 (28.2)		23 (59.0)		39 (22.4)	
Unlisted	3 (10.7)		10 (35.7)		15 (53.6)		28 (16.1)	
Source of injury								
Work-related	14 (28.0)		16 (32.0)		20 (40.0)		50 (28.7)	
Motor vehicle accident	7 (11.5)		22 (36.1)		32 (52.5)		61 (35.1)	
Other	1 (33.3)		0 (0)		2 (66.7)		3 (1.7)	
Unlisted	12 (20.0)		22 (36.7)		26 (43.3)		60 (34.5)	
	M	SD	M	SD	M	SD	M	SD
Age	44.44	7.76	44.79	8.82	47.38	9.82	45.85	9.09
Time off work (months)	26.94	11.51	51.79	68.93	26.35	28.06	35.87	47.28
Years of education	13.44	2.87	13.18	4.03	13.48	3.88	13.36	3.73
Pain duration (months)	47.61	29.25	68.26	62.01	46.40	41.1	54.72	48.85
Number of injuries	2.83	2.09	2.41	1.67	2.65	2.68	2.60	2.22

Prior to analysis, data on the psychosocial measures were explored to identify whether any missing values existed; it was discovered that 43 participants were missing between 0.6% and 11.6% of data across 10 of the 21 dependent variables. These missing values were filled using a multiple imputation procedure in SPSS to retain the original sample size and to produce valid statistical inferences.

The descriptive statistics for each time point and the results of the mixed ANOVAs are displayed in Table 2. Significance was found for CES-D (F = 17.63, *P* < 0.001); PCS (F = 11.37, *P* < 0.001); CAS (F = 17.63, *P* < 0.001), PRIME-MD PQ (F = 7.76, *P* = 0.001); the CPAQ subscale of Activity Engagement (F = 29.24, *P* < 0.001); the PSOCQ subscales of Precontemplation (*F =* 10.82, *P* < 0.001), Contemplation (F* =* 5.62, *P *= 0.002), and Maintenance (F* =* 16.60, *P *< 0.001); and the CPCI subscales of Task Persistence (F = 6.30, *P *= 0.002), Pacing (F = 6.70, *P *= 0.002), and Seeking Social Support (F* =* 6.70, *P *= 0.002). Though significance was not reached for the PDI (F = 5.62, *P* = 0.004) and the Action subscale of the PSOCQ (F = 5.62, *P* = 0.004), both *P* values were closely approaching significance and thus warrant further exploration.

**Table 2.** Descriptive statistics and mixed ANOVA results for dependent measures by group.

		Poorly–fairly		Well		Very well–excellent			
Measure	Time^a^	M	SE	M	SE	M	*SE*	F	Sig.
PIS	1	6.97	0.29	6.05	0.22	6.19	0.19	1.10	0.336
	2	6.41	0.59	6.01	0.45	6.25	0.39		
CES-D	1	39.15	1.88	33.27	1.41	30.13	1.22	17.63*	0.000
	2	34.03	1.93	25.07	1.45	19.71	1.26		
PCS	1	35.29	2.05	29.68	1.54	27.24	1.34	11.37*	0.000
	2	29.79	2.00	22.90	1.51	17.44	1.31		
CAS	1	47.25	2.95	38.73	2.22	31.41	1.92	18.39*	0.000
	2	46.28	3.09	34.71	2.32	23.17	2.01		
PRIME-MD PQ	1	13.89	0.66	12.60	0.49	12.63	0.43	7.76*	0.001
	2	14.24	0.70	11.60	0.52	9.85	0.45		
PDI	1	49.38	1.65	46.15	1.24	46.34	1.07	5.62	0.004
	2	48.79	1.71	44.23	1.28	40.04	1.11		
CPAQ									
Activity Engagement	1	17.09	1.70	22.55	1.28	27.55	1.11	29.23*	0.000
	2	20.74	1.56	30.25	1.18	35.37	1.02		
Pain Willingness	1	16.94	1.46	18.93	1.10	19.04	0.95	2.42*	0.092
	2	17.12	1.17	20.40	0.88	20.89	0.77		
PSOCQ									
Precontemplation	1	2.97	0.11	2.72	0.09	2.65	0.07	10.82*	0.000
	2	2.74	0.11	2.40	0.08	2.00	0.07		
Contemplation	1	3.88	0.09	4.01	0.07	4.13	0.06	6.31*	0.002
	2	3.77	0.08	3.93	0.06	4.14	0.06		
Action	1	3.32	0.13	3.47	0.09	3.48	0.08	5.62	0.004
	2	3.74	0.10	3.97	0.07	4.28	0.06		
Maintenance	1	3.00	0.13	3.13	0.10	3.24	0.08	16.60*	0.000
	2	3.44	0.09	3.92	0.07	4.33	0.06		
CPCI									
Guarding	1	56.47	1.20	53.38	0.90	53.60	0.78	3.50	0.032
	2	54.85	1.25	51.88	0.94	50.96	0.81		
Resting	1	55.24	1.54	54.87	1.16	52.90	1.00	1.24	0.292
	2	54.71	1.25	54.75	0.94	53.49	0.81		
Asking for Assistance	1	53.53	1.50	52.85	1.13	51.93	0.98	0.28	0.759
	2	52.53	1.45	51.00	1.09	51.88	0.94		
Exercise/Stretch	1	49.85	1.67	49.58	1.25	50.54	1.09	1.75	0.177
	2	54.79	1.36	57.57	1.03	59.50	0.89		
Relaxation	1	49.41	1.58	48.52	1.19	51.10	1.03	3.32	0.038
	2	58.09	1.39	60.73	1.05	63.00	0.91		
Task Persistence	1	36.77	1.62	41.98	1.22	43.11	1.06	6.30*	0.002
	2	36.32	1.23	39.15	0.93	40.23	0.81		
Coping Self-Statements	1	46.00	1.55	47.83	1.17	49.89	1.01	3.70	0.027
	2	47.88	1.45	50.85	1.09	52.70	0.94		
Pacing	1	47.35	1.27	49.67	0.96	50.40	0.83	6.70*	0.002
	2	51.03	1.19	53.48	0.90	56.74	0.78		
Seeking Social Support	1	47.91	1.49	49.88	1.12	51.60	0.97	6.70*	0.002
	2	46.88	1.48	51.13	1.11	54.90	0.97		

^a^Time: 1 = admission; 2 = discharge

*F statistic is significant at the 0.002 level.

ANOVA = analysis of variance; PIS = Pain Intensity Scale; CES-D = Center for Epidemiological Studies–Depressed Mood Scale; PCS = Pain Catastrophizing Scale; CAS = Clinical Anxiety Scale PRIME-MD PQ = Patient Questionnaire of the Primary Care Evaluation of Mental Disorders; PDI = Pain Disability Index; CPAQ = Chronic Pain Acceptance Questionnaire; PSOCQ = Pain Stages of Change Questionnaire; CPCI = Chronic Pain Coping Inventory.

Analyses of the differences were conducted with post hoc pairwise Bonferroni tests to determine where the difference was on each psychosocial factor. In SPSS, the Bonferroni correction is automatically applied, so results can be interpreted at the 0.05 significance level. The results of these tests, which include the mean differences of each comparison and significance levels, are displayed in Table 3.

**Table 3.** Results of post hoc Bonferroni pairwise comparisons.^a^

Measure	(I) Groupings vs. (J) groupings		Mean difference (I − J)	SE	Sig.
PIS	Very well–excellent	Poorly–fairly	−0.47	0.43	0.818
	Very well–excellent	Well	0.19	0.36	1.000
	Well	Poorly–fairly	−0.66	0.45	0.428
CES-D	Very well–excellent	Poorly–fairly	−11.67*	1.97	0.000
	Very well–excellent	Well	−4.25*	1.65	0.032
	Well	Poorly–fairly	−7.42*	2.07	0.001
PCS	Very well–excellent	Poorly–fairly	−10.21*	2.15	0.000
	Very Well–Excellent	Well	−3.95	1.80	0.087
	Well	Poorly–fairly	−6.25*	2.26	0.019
CAS	Very well–excellent	Poorly–fairly	−19.48*	3.28	0.000
	Very well–excellent	Well	−9.43*	2.74	0.002
	Well	Poorly–fairly	−10.05*	3.44	0.012
PRIME-MD PQ	Very well–excellent	Poorly–fairly	−2.83*	0.72	0.000
	Very well–excellent	Well	−0.86	0.60	0.457
	Well	Poorly–fairly	−1.97*	0.75	0.029
PDI	Very well–excellent	Poorly–fairly	−5.90*	1.76	0.003
	Very well–excellent	Well	−2.00	1.47	0.524
	Well	Poorly–fairly	−3.90	1.85	0.109
CPAQ					
Activity Engagement	Very well–excellent	Poorly–fairly	12.55*	1.66	0.000
	Very well–excellent	Well	5.06*	1.38	0.001
	Well	Poorly–fairly	7.49*	1.74	0.000
Pain Willingness	Very well–excellent	Poorly–fairly	2.94	1.38	0.103
	Very well–excellent	Well	0.30	1.15	1.000
	Well	Poorly–fairly	2.64	1.44	0.209
PSOCQ					
Precontemplation	Very well–excellent	Poorly–fairly	−0.53*	0.12	0.000
	Very well–excellent	Well	−0.23*	0.10	0.049
	Well	Poorly–fairly	−0.29*	0.12	0.048
Contemplation	Very well–excellent	Poorly–fairly	0.31*	0.09	0.002
	Very well–excellent	Well	0.16	0.08	0.105
	Well	Poorly–fairly	0.15	0.09	0.355
Action	Very well–excellent	Poorly–fairly	0.35*	0.11	0.004
	Very well–excellent	Well	0.16	0.09	0.227
	Well	Poorly–fairly	0.19	0.11	0.265
Maintenance	Very well–excellent	Poorly–fairly	0.56*	0.10	0.000
	Very well–excellent	Well	0.26*	0.08	0.007
	Well	Poorly–fairly	0.30*	0.10	0.012
CPCI					
Guarding	Very well–excellent	Poorly–fairly	−3.38*	1.32	0.033
	Very well–excellent	Well	−0.35	1.10	1.000
	Well	Poorly–fairly	−3.03	1.38	0.089
Resting	Very well–excellent	Poorly–fairly	−1.78	1.43	0.650
	Very well–excellent	Well	−1.61	1.20	0.536
	Well	Poorly–fairly	−0.16	1.50	1.000
Asking for Assistance	Very well–excellent	Poorly–fairly	−1.13	1.61	1.000
	Very well–excellent	Well	−0.02	1.34	1.000
	Well	Poorly–fairly	−1.10	1.69	1.000
Exercise/Stretch	Very well–excellent	Poorly–fairly	2.70	1.51	0.226
	Very well–excellent	Well	1.44	1.26	0.756
	Well	Poorly–fairly	1.25	1.58	1.000
Relaxation	Very well–excellent	Poorly–fairly	3.30	1.47	0.077
	Very well–excellent	Well	2.42	1.22	0.147
	Well	Poorly–fairly	0.88	1.54	1.000
Task Persistence	Very well–excellent	Poorly–Fairly	5.12*	1.45	0.002
	Very well–excellent	Well	1.10	1.21	1.000
	Well	Poorly–fairly	4.02*	1.52	0.027
Coping Strategies	Very well–excellent	Poorly–fairly	4.35*	1.63	0.025
	Very well–excellent	Well	1.95	1.36	0.459
	Well	Poorly–fairly	2.40	1.71	0.486
Pacing	Very well–excellent	Poorly–fairly	4.38*	1.22	0.001
	Very well–excellent	Well	1.99	1.02	0.155
	Well	Poorly–fairly	2.38	1.28	0.192
Seeking Social Support	Very well–excellent	Poorly–fairly	5.85*	1.64	0.001
	Very well–excellent	Well	2.74	1.37	0.139
	Well	Poorly–fairly	3.11	1.72	0.215

^a^The error term is mean square(error) = 63.890.

*The mean difference is significant at the 0.05 level.

ANOVA = analysis of variance; PIS = Pain Intensity Scale; CES-D = Center for Epidemiological Studies–Depressed Mood Scale; PCS = Pain Catastrophizing Scale; CAS = Clinical Anxiety Scale PRIME-MD PQ = Patient Questionnaire of the Primary Care Evaluation of Mental Disorders; PDI = Pain Disability Index; CPAQ = Chronic Pain Acceptance Questionnaire; PSOCQ = Pain Stages of Change Questionnaire; CPCI = Chronic Pain Coping Inventory.

For the CES-D, all groups were significantly different from one another; the very well–excellent group improved more than the well group and the poorly–fairly group and the well group improved more than the poorly–fairly group. For the PCS, CAS, PRIME-MD PQ, and the Activity Engagement subscale of the CPAQ, the very well–excellent group improved more than the poorly–fairly group, and the well group also improved more than poorly–fairly group on each of these scales. For the PDI, the very well–excellent group improved more than the poorly–fairly group.

For each of the Precontemplation and Maintenance subscales of the PSOCQ, all groups were significantly different from one another; the very well–excellent group improved more than both the well group and the poorly–fairly group and the well group improved more than the poorly–fairly group. On each of the Contemplation and Action subscales, the very well–excellent group improved more than the poorly–fairly group.

For the Task Persistence, Pacing, and Seeking Social Support subscales of the CPCI, the very well–excellent group improved more than the poorly–fairly group. In addition, on the Task Persistence subscale, the well group improved more than the poorly–fairly group.

## Discussion

This study sought to understand the treatment-related changes in psychosocial factors that correspond to patients’ perceptions of global self-improvement after attending a chronic pain management program. Results from this study showed that participants’ self-reported improvement levels differed significantly on experiences of emotional distress (i.e., depression, anxiety, catastrophizing), general health, activity engagement, three of the four stages of readiness to change (i.e., precontemplation, contemplation, and maintenance), and three adaptive coping strategies (i.e., task persistence, pacing, and seeking social support); the differences between groups in improvement approached significance on the action stage of readiness to change and on pain-related disability. Further comparisons demonstrated that those who rated themselves highly (very well–excellent) in terms of having accomplished their goals also showed greater improvements on the above measures than those who rated their improvement to be poor (poorly–fairly). Taken together, these findings suggest that patients required a large magnitude of improvement in the above stated experiences in order to rate themselves as highly improved overall, as opposed to those who rated their self-improvement as poor or unsuccessful, because they demonstrated much lower magnitudes of improvement in these experiences.

Reductions in experiences of emotional distress, pain-related disability (approaching significance), and negative health were associated with higher ratings of improvement for participants, and these associations are consistent with previous findings.^16,20^ Furthermore, particularly for experiences of depression, the very well–excellent group demonstrated greater improvements than the well group, and both groups expressed greater improvements than the poorly–fairly group. The cascading effect recognized in ratings of depression across groups suggests that though a certain magnitude of reduction in depressive symptoms may be required for feeling content (well) about one’s improvement, an even greater magnitude of reduction is required for these patients to transition to feeling strongly improved after chronic pain treatment.

Though differences between groups on the magnitude of improvement they reported regarding pain-related disability did not reach significance, the *P* value was close enough to warrant further exploration. A reduction in pain-related disability can indicate that the participant has a low perception of pain’s interference with his or her daily activities. Lower pain-related disability could be a result of the development of higher self-efficacy; that is, development of a stronger perception of one’s ability to manage their chronic pain, complete day-to-day tasks, and accomplish their goals.^42^ This is consistent with previous literature that found that high pain control and high self-efficacy are associated with lower perceived disability and lower levels of pain interference.^2,3,43,44^ These higher self-efficacy beliefs may have manifested in two ways: through receipt of education regarding management of one’s pain and through performance of tasks throughout the program and witnessing one’s success in them. As suggested by Fox's skill enhancement hypothesis,^45^ successful performance in tasks helps people reinforce positive perceptions about themselves, and according to the self-enhancement hypothesis, people seek out and engage in tasks in which they can develop positive perceptions about their abilities. As per these hypotheses, participants who engage in tasks successfully will then develop greater confidence in their abilities to perform, despite their pain-related adversity. Thus, they would be more likely to engage in behaviors related to improvement in their situation, which would result in an upward cycle of success and engagement toward the achievement of their goals. Programming that emphasizes the enhancement of one’s perceptions of one’s competencies and abilities can lead to the adoption of positive beliefs and behaviors related to one’s improvement.

It was recognized that participants in this study all required significant improvements in factors of activity engagement, task persistence, and pacing to rate their global self-improvement strongly. These factors are related to one another in that they are approaches to promote engagement in tasks and activities. Activity engagement relates to the extent to which one involves him or herself in a task despite pain; task persistence relates to the extent one continues this involvement; and pacing involves use of strategy that allows engaging in an activity in a gradual manner with intermittent rest or slowdown periods in order to persist in that activity over longer periods of time.^22^ The importance of these task-oriented factors hints at the idea that patients value treatment that helps them learn to perform and persist through activities despite pain and that patients may find it important for their overall treatment to help them continue daily functioning. As suggested previously, with a greater repertoire of coping strategies and acceptance of pain (through activity engagement), these approaches may help participants gain higher self-efficacy to perform and persist through tasks and thus reduce perceptions of pain-related disability. This is in line with previous literature that has found acceptance to be complementary to adaptive coping self-management approaches^22^ and is associated with less pain-related disability. Furthermore, in line with this study’s findings, the literature suggests that activity engagement is powerfully related to depression, anxiety, and pain interference.^46^

Seeking social support as a coping strategy has been associated with less pain in chronic pain participants.^47^ The resulting improvements may be regarded as positive outcomes of attendance in a group environment; in a study by Subramaniam et al.,^48^ participants who attended programs in groups displayed better improvement than those who received individual treatment, which was likely due to the collective feedback and mutual support they received from their peers. Support, in the form of feedback, can lead to internalization of positively associated behaviors^49^; support and feedback received from others may have led to participants’ internalizing and adopting these positive supports as their own values and beliefs and thus report themselves as more improved in comparison to those who do not attain feedback or seek support. By encouraging group interaction and providing mutual support to group members, as well as informing families and friends to do the same, a participant may improve more in the program and rate his or her improvement higher.

Differences were recognized between groups on the magnitude of change they reported on the subscales of the PSOCQ (i.e., precontemplation, action [approaching significance], and maintenance), which measures readiness to change. These findings were in line with previous research that identified improvements in readiness to change related to overall improvement in perceptions.^21^ Readiness to change lies on a continuum in which each stage captures a certain set of attitudes and behaviors that become more adaptive as one transitions forward across each stage. The magnitudes of change may be more profound after engaging in treatment, because patients who may previously have been categorized into the precontemplation or contemplation stage have likely transitioned to action and maintenance stages as they adopted a self-management approach. Furthermore, movement through these stages may be catalyzed by the learning, application, and adoption of adaptive coping strategies,^50^ which patients reported improved use of in this study.

This study was not without its limitations. A strength of the Self-Evaluation Scale was that it suggests universal criteria for rating one’s improvement (e.g., improve general health and nutrition, reduction in medications), so that participants may be more inclined to rate themselves on these treatment-related goals, rather than on domains of improvement that may not be related to the factors addressed by the chronic pain management program. However, the domains suggested in this scale may not be domains that participants find personally important. To address this issue, a comment section is also included in the scale for the participant to elaborate on and add justification for the rating that he or she provided.^51^

All of the questionnaires concerning the psychosocial factors of the program have been tested for reliability, validity, and sensitivity to change. However, possible floor and ceiling effects were not accounted for. For instance, for participants who may have already reported high scores on the measures such as Activity Engagement and have genuinely improved on this factor throughout the period of treatment, a report of a high score at discharge may not reflect the actual magnitude of change they experienced on that factor. This bias would also be present for those who scored low on measures such as the CES-D. This is particularly limiting on questionnaires that involve transition through stages such as the PSOCQ, in which a participant who initially indicated a high score on the Precontemplation subscale and a lower score after treatment would demonstrate a greater magnitude of change than a participant who scored low on the same subscale at both time points. To address these issues, future studies could consider retrospective questionnaires, in which participants rate at the second time point how they felt before treatment to how they feel after treatment. This method has demonstrated greater validity at identifying change than traditional pre–post questionnaires.^52^ In addition, some of the measures such as the CES-D and CAS attempted to evaluate global mental states, in which the items in these measures are not pain specific or necessarily pain relevant. If items were more pain specific, their associations with one’s perceptions of improvement may have been more significant in this study if their depressive, anxious, or happy states are influenced by the pain they endure. For example, participants who reported a greater level of global improvement may have reported greater improvements in pain-specific depression than those who reported lower levels of improvement, as opposed to reporting improvements in global experiences of depression. Finally, the measures also varied in the time frames used when asking participants to assess their experiences. For instance, the PRIME-MD PQ asked participants to assess symptoms over a period of 4 weeks, whereas the PIS asked participants to assess severity in the past 2 weeks. This introduces variance in the findings; future studies could address this by ensuring consistency in time frames that are proposed across all measures.

Future work could look to evaluate the expectations that the participants have of their improvement prior to the program and compare these expectations to their perceptions of self-improvement after treatment. It would be interesting to see whether a patient who projects great improvement and satisfaction in the beginning of the program improves more than those who do not. In addition, providers can utilize the perceived expectations to help create individual goals and realistic expectations that are reachable for the participant; this involvement of the patient in planning his or her treatment based on his or her expectations could help lead to positive perceptions of self-improvement.

## Conclusions

This study identified some of the psychosocial factors that may be most valued or important to participants in their perceptions of self-improvement. It was discovered that for patients to perceive their improvement as successful as opposed to unsuccessful, they required a greater extent of improvement across multiple domains that relate to improving one’s ability to engage and persist through tasks and reducing experiences of emotional distress and disability. Insights from this study can help inform patient-centered care in addressing the needs of participants as they relate to these valued psychosocial factors by adopting treatment approaches that address the multidimensional nature of the patient’s experience. This understanding can also enhance communication in patient–provider relationships about what criteria can be involved in success for patients to help them create goals, informed choices, and realistic expectations.
